# An Inexpensive, Fast and Sensitive Quantitative Lateral Flow Magneto-Immunoassay for Total Prostate Specific Antigen

**DOI:** 10.3390/bios4030204

**Published:** 2014-07-08

**Authors:** Jacqueline M. Barnett, Patrick Wraith, Janice Kiely, Raj Persad, Katrina Hurley, Peter Hawkins, Richard Luxton

**Affiliations:** 1 Institute of Bio-Sensing Technology, University of the West of England, Bristol, BS16 1QY, UK; E-Mails: jackie.barnett@uwe.ac.uk (J.M.B.); patrickwraith@blueyonder.co.uk (P.W.); janice.kiely@uwe.ac.uk (J.K.); hp@hphawkins.com (P.H.); 2 Bristol Urological Institute, Southmead Hospital, Bristol, BS10 5NB, UK; E-Mail: rajpersad@bristolurology.com; 3 Department of Surgery, University Hospitals Bristol NHS Trust, Upper Maudlin Street, Bristol, BS2 8HW, UK; E-Mail: Katrina.Hurley@uhbristol.nhs.uk

**Keywords:** paramagnetic particles, resonant coil magnetometer, prostate specific antigen

## Abstract

We describe the detection characteristics of a device the Resonant Coil Magnetometer (RCM) to quantify paramagnetic particles (PMPs) in immunochromatographic (lateral flow) assays. Lateral flow assays were developed using PMPs for the measurement of total prostate specific antigen (PSA) in serum samples. A detection limit of 0.8 ng/mL was achieved for total PSA using the RCM and is at clinically significant concentrations. Comparison of data obtained in a pilot study from the analysis of serum samples with commercially available immunoassays shows good agreement. The development of a quantitative magneto-immunoassay in lateral flow format for total PSA suggests the potential of the RCM to operate with many immunoassay formats. The RCM has the potential to be modified to quantify multiple analytes in this format. This research shows promise for the development of an inexpensive device capable of quantifying multiple analytes at the point-of-care using a magneto-immunoassay in lateral flow format.

## 1. Introduction

Magnetic particles have many applications in biological research and are used very successfully as a solid phase to aid separation [1]. Examples include the removal of tumour cells from bone marrow, isolation of T-cells, viruses and organelles. In immunoassays magnetic particles are used extensively as a solid phase e.g., Immulite Diagnostics Products Corporation, Siemens, Erlangen, Germany; FastPack, Qualigen, Carlsbad, CA and in molecular genetics in various kits for the isolation of nucleic acid. Recently attention has focused on using magnetic particles, ferromagnetic (permanently magnetized) or paramagnetic particles (PMP) (magnetic only when in a magnetic field), as labels that are detected directly by virtue of their magnetic properties. Magnetic particles have a number of advantages in use as labels in immunoassays including their stability and that they are not affected by the sample matrix. These properties are particularly suitable for labels used in the development of point-of-care tests. 

Paper-based point-of-care diagnostics including lateral flow assays are affordable, user friendly, rapid, robust and scalable for manufacturing. Lateral flow assays have a large potential for use by unskilled personnel in a wide variety of settings including those that are resource-limited [2]. Lateral flow devices have been previously modified such that a wide variety of sample types can be evaluated. In addition the automation of production is well supported and cost of test strips is very low. This makes the combination of a lateral flow assays for use with magnetic particles an attractive proposition for development.

Lateral flow assays have been quantified using a range of techniques [3]; however optical methods have a disadvantage in that they measure only those particles present on the membrane surface. In contrast the Resonant Coil Magnetometer (RCM) will measure particles present in the antibody (Ab) capture line throughout the membrane. The results are therefore truly quantitative of the whole test not only the proportion detected on the surface of the LF strip. Additional advantages are that the label is stable and therefore LF strips can be reanalyzed if required. For chemiluminescent labels the label is consumed when the chemical reaction takes place so no further measurements are possible. Although fluorescent labels can be re-stimulated and multiple measurements made in a short time period to improve the measurement signal to noise ratio, many fluorescent labels decompose on prolonged re-stimulation because of photo-bleaching. 

A range of sensors have been developed by commercial and research groups to quantify magnetic particles for example MagnaBioSciences, LLC, Quantum Design, San Diego, CA, USA [4], LifeAssays AB, Lund, Sweden [5], Senova Immunoassay Systems, Jena, Germany [6], Magnotech, Philips, Amsterdam, The Netherlands [7], Magnisense, Paris, France [8] and MagArray Inc., Sunnyvale, CA, USA [9]. A number of Giant Magnetoresistance based (GMR) sensors are being developed as fully integrated systems which can detect a small number of PMPs [10]. The RCM measurements are produced from the interaction of many PMPs; this means that the results will not be influenced by variability in PMP size or magnetite concentration that devices that measure few particles could be susceptible to [11]. In addition only PMPs that are non-aggregated can flow through nitrocellulose membranes and give results at the test and control line. In contrast PMPs in solution will still give results, however the data may be variable dependent on the level of aggregation present and therefore this needs to be monitored, particularly during conjugation to the biological recognition element. A review of GMR sensors in development [12] describes the use of PMPs 40–2800 nm by this technique and the need to maintain PMPs in a mono-dispersed non-aggregated state. The data show a relatively narrow range of size distribution; however the presence of even doublet or triplet PMPs may cause variability in results. In CMOS Hall sensors the resulting bead signal is very small; this is managed by the use of miniaturized Hall-effect sensors which can be prone to problems from fabrication imperfections such as contact misalignment [13]. For GMR sensors the sensing spot has a very small footprint and can detect single beads if they are very closely connected with the sensor. This would be difficult to achieve for a relatively widespread line of PMPs diffused through the lateral flow strip. Our, relatively simple and cheaper arrangement measures the changes in a perturbed high frequency inductive field, and does not have to be so precisely aligned, making the mechanics less critical and expensive. 

The RCM is capable of detecting PMPs of a wide range of sizes and commercial suppliers from 2.8 µm to 0.250 µm [14]. GMR and Hall sensors can also detect a range of PMP sizes but individual sensors cannot detect the full range of PMPs, the RCM therefore has flexibility with the size of PMP used [14]. As the RCM is not fully integrated this device also has flexibility in the type of immunoassay conducted. The main advantages of the RCM device in comparison with GMRs and CMOS Hall sensors is in the speed of the assay time, with RCM measurements being collected over 1–2 min [14] the cost of the device, with component parts estimated at approximately $100 and the applicability of the sensor to be a hand-held device [11]. A detailed review of the merits of different magnetic sensors is described elsewhere [10,11].

Much debate exists over the usefulness of prostate specific antigen (PSA) as a biomarker for the detection of prostate cancer, however the data suggest that it can provide useful information in assisting in the diagnosis of this disease with PSA concentrations 4–10 ng/mL having a 25% positive predictive value, to greater than 80% in men with a PSA >20 ng/mL [15]. In this study we investigate the development of magneto-immunoassays in lateral flow format with PMPs as labels to quantify total prostate specific antigen (PSA^T^). The aim of this investigation is to demonstrate that the RCM can be used to quantify analytes in immunoassays in lateral flow format giving the potential for performing PSA^T ^ assays in the doctors’ surgery.

The RCM is a sensor developed at the University of The West of England to quantify PMPs [14,16], This sensor has the potential to be developed as an inexpensive device for use in the field or at the point-of-care. The RCM is based on a Frequency Locked Loop design. The original observations reported an RCM with a helical coil, further work with oval and rectangular coils [17] showed a direct correlation between number of PMPs and decrease in the measured resonant frequency. It has also been shown experimentally that a single hand-wound flat spiral coil produces a linear decrease in resonant frequency when an increasing number of PMPs are placed next to it [18].

From the formula shown below Equation (1), for a resonant tuned circuit that is tuning a Voltage Controlled Oscillator (VCO), it can be seen that the higher the resonant frequency (F), and the smaller the inductance of the coils (L) in relation to the value of the capacitance (C), very small changes in inductance due to PMPs would result in large changes in frequency.

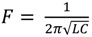
(1)


**Figure 1:**
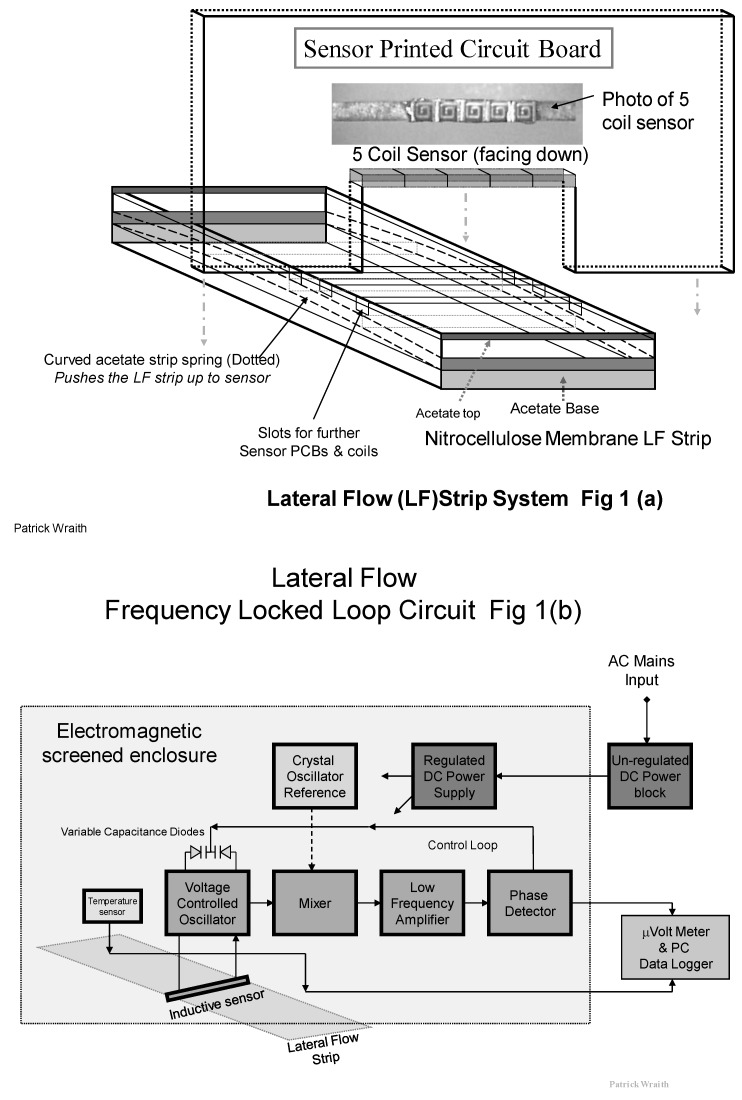
(**a**) Diagram of the five coil surface mount sensor and its location and assembly in the Resonant Coil Magnetometer (RCM); (**b**) Diagram of the Phase Locked Loop (FLL) circuit used with the RCM with the inductive sensor comprised of an array of five flat spiral coils.

The prototype RCM for use with lateral flow assays used in this study is based upon a **F**requency **L**ocked **L**oop circuit shown in Figure 1b, where the Very High Frequency (VHF) Voltage Controlled Oscillator (VCO) is pulled into frequency lock with a crystal controlled fixed reference signal. An inductive sensor forms the tuning inductance of the VCO along with associated. The crystal reference oscillator produces a very stable signal at 60 MHz. This VCO signal at 59.94 MHz and the reference signals at 60 MHz are mixed together at VHF to produce a very **L**ow difference **F**requency of 60 KHz. This LF contains the same variations as those produced by the small changes in inductance at VHF due to the PMPs. If a sample strip with paramagnetic particles influences the sensor, the field permeability of the associated field and the sensor inductances are very slightly increased. This tends to decrease the VCO frequency, resulting in a corresponding increase in the LF. A positive Direct Current (DC) error voltage is produced from the phase detector, and this DC is applied back to the variable-capacitance diodes to increase the VCO frequency back to normal lock with the crystal reference. This frequency correction is done within milliseconds and the Frequency Locked Loop is continuously tracking the crystal reference. It is the change in DC that is a measure of the PMPs present on the test and control lines in the nitrocellulose membrane strip and is measured on a Milli-Voltmeter and data logger.

The sensitivity of this system is such that the frequency changes occurring at the **R**adio **F**requency around 60 MHz are only of a few KHz or less. This is a change of frequency in the order of only 0.0016% and is difficult to detect By converting the RF to a very low frequency using a Radio Frequency Mixer stage to heterodyne the crystal Reference and the VCO frequencies together, the (low) difference frequency, applied to a phase (PD) detector circuit, produces a DC error voltage that delivers the measured sensor output changes relative to the quantity of PMPs. 

The frequency and phase shift is now 1000 times larger in relation to the LF, than it was at VHF *i.e*., 1.6%, thus greatly improving the signal to noise ratio of the electrical signals, and improving the possible detection resolution. The low frequency signal is also amplitude limited prior to the Phase Detector, to remove any variations in signal strength and remove impulse noise before detection. This is further improves on the detection resolution. This magnetic sensor is sensitive to changes in the order of femto-henries. There may be small temperature fluctuations due to dimensional changes and for this reason a temperature sensor is mounted nearby in order to create a DC reference which changes in proportion to this variation and provides a more temperature stable differential voltage measurement to the micro-voltmeter and logging Personal Computer (PC). 

The VCO frequency stability is sensitive to supply variations, so a DC voltage regulator circuit is included to stabilize the supply from an external AC to DC Mains power block. 

The Frequency Locked Loop (FLL) circuit is built on a vertically mounted Printed Circuit Board (PCB) such that the inductive sensor coils are located across the top of the strip. See Figure 1a. The inductance (L) of the resonant VCO tuned circuit is shared between five tiny surface mount inductors, rather than a single coil, to spread the field pattern across the capture line of PMPs.

The strip is inserted into the rectangular slide, and a flattened loop of acetate strip is used as a spring to push the PMP capture line upward onto the lateral flow strip into critically precise proximity to the inductive field from the surface mount coils. This design of sensor produces a rectangular “curtain” sense field which closely and efficiently matches the width and length of the PMP line. 

As a result of this “curtain field” pattern the RF radiation of the field does not extend far outward along the strip. This arrangement may allow us to co-locate duplicate FLL sensor circuits along the strip for simultaneous measurement from other capture lines such as for reference purposes, without RF interference between the circuits. A diagram showing the RCM design used in this study is shown in Figure 1a.

## 2. Experimental Section

### 2.1. Materials

PMPs are available from several manufacturers in a range of sizes from a few nanometers to several microns. The PMPs used throughout this study were 760 nm carboxylic acid modified particles obtained from Seradyn Inc., Indianapolis, IL, USA. The primary protein layer used to prepare the PMP conjugate was goat anti-mouse antibody purchased from Dako, UK, Ltd., Ely. Monoclonal antibodies for total PSA (total PSA capture antibody, PSAT^CAPTURE^ and PSA detecting antibody PSAT^DETECTION^) were supplied as a gift by Randox Laboratories Ltd. (Crumlin, County Antrim, Northern Ireland). The control line rabbit anti-mouse antibody was purchased from Dako Ltd., Ely, Cambridgeshire, UK.

Lateral flow assays for total PSA were calibrated with the WHO international standard preparation of total PSA 96/670 (PSA^T^), National Institute for Biological Standards and Control (NIBSC), Potters Bar, Hertfordshire, UK. Twenty five serum samples from anonymous patients were obtained from a urology out-patients clinic (Bristol Royal Infirmary), stored at 4 °C and tested within two weeks in the magneto-immunoassay for total PSA. Approval was obtained from the Central and South Bristol Ethics Committee (05/Q2006/57) and from the UWE (University of The West of England, Bristol, UK) ethics committee (221) to enable the collection and testing of the serum samples. Control HIV negative human AB male serum was purchased from BioWest Ltd., East Sussex, UK. 

The Immulite total PSA assay (Catalogue number L2KPS6) was performed on an Immulite 2000 Analyser (Diagnostic Products Corporation, DPC, Los Angeles, CA, USA). This is a sandwich immunoassay where the solid phase bound complex is detected by the addition of a chemiluminescent substrate, measured using the Immulite 2000 Analyzer.

The FastPack PSA assay is a sandwich immunoassay with a chemiluminescent label where antibody coated PMPs are used to facilitate the washing steps. The label is quantified using the FastPack bench top analyser (Qualigen Inc, Carlsbad, CA, USA).

### 2.2. Resonant Coil Magnetometer (RCM)

Voltage readings in millivolts (mV) were taken with the surface mounted resonant coil magnetometer (RCM) using a voltage meter (Thurby Thander Instruments, S.J. Electronics, Telford Way Industrial Estate, Kettering, Essex, UK). Magnetometer readings were taken every ten seconds for 100 s for each membrane without PMPs and every ten seconds for 100 s with PMPs at the test line and control line.

### 2.3. Deposition of Naked Particles on Nitrocellulose Membranes

Strips with a range of Seradyn 760 nm PMP dilutions were prepared with a BioDot AD3200 (BioDot Ltd., Kingley Centre, Chichester, UK) by non-contact deposition. PMPs were dispensed at a rate of 1.7969 µL/cm. Calibration curves were produced by plotting particle numbers against change in voltage (mV) measured using the RCM.

### 2.4. Lateral Flow Assay for Total PSA and Free PSA

**Figure 2:**
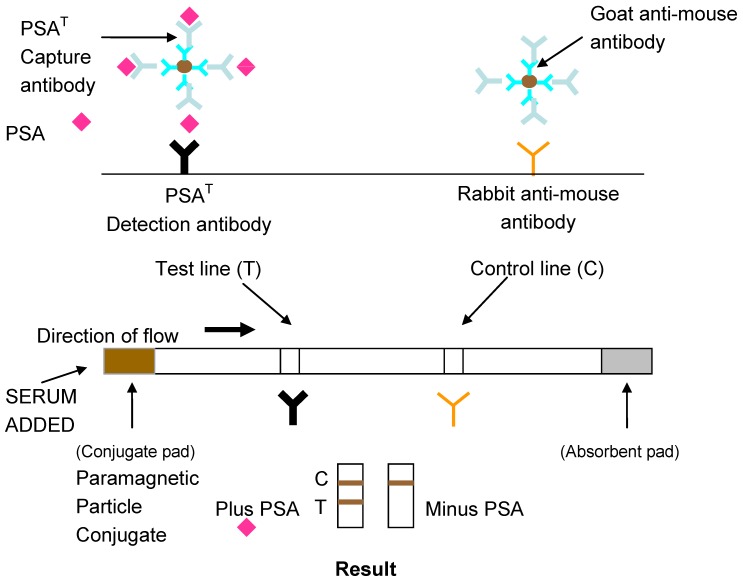
Lateral flow Magneto-immunoassay for Total or Free Prostate Specific Antigen (PSA).

The development of lateral flow assays to detect specific epitopes of an antigen is possible with the use of monoclonal antibodies to prepare the conjugate and antibody capture lines. A layer of anti-mouse antibody was absorbed on the conjugate to ensure the correct orientation of the capture antibody on the PMPs. Previous work has shown this to increase the detection sensitivity of lateral flow assays [19]. A schematic diagram of the design of the lateral flow assay for total PSA using monoclonal antibodies is shown in Figure 2.

**Table:** 

Key:	
PSA^T^ Capture antibody	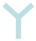
Goat anti-mouse antibody	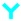
PSA^T^ Detection antibody	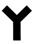
Rabbit anti-mouse antibody	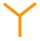
Paramagnetic particle conjugate	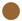
Prostate specific antigen	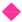

#### 2.4.1. Antibody Conjugation to PMP

The passive adsorption of monoclonal antibody to PMPs was carried out as a two-step process using an adaptation of a protocol for passive adsorption described by Interfacial Dynamics (IDC, Molecular Probes Inc., Eugene, OR, USA). All washes were performed using a Dynal magnetic separator (Dynal MPC-S, Dynal Biotech UK, Bromborough, Wirral). Firstly goat anti-mouse IgG (Dako, UK, Ltd., Ely) was adsorbed. The PMPs (5 mg containing approximately 1 × 10^9^ PMPs) were washed twice using the 1 mL of 0.025 M MES (2-[N-Morpholino] ethanesulfonic acid buffer), (Sigma-Aldrich, Poole, Dorset), pH 6.3 and resuspended in 0.5 mL of the same buffer. The PMP solution was sonicated with three bursts of 2 s; setting 6 of a MSE Soniprep 150 sonicator (Henderson Biomedical Ltd., Lower Sydenham) to ensure that the particles were mono-dispersed. Goat anti-mouse IgG, 40 µL (equivalent to 56 µg) was then added to the particles in 0.5 mL of 0.025 M MES buffer pH 6, the solution was then incubated at room temperature (RT) overnight with continuous mixing.

The supernatant was removed and the PMPs were washed twice with 1 mL of 0.025 M MES buffer pH 6.3 (Sigma-Aldrich, Poole, Dorset) and resuspended in 0.5 mL of the same buffer. The supernatant, containing excess anti-mouse IgG and 50 µL of the resuspended goat anti-mouse conjugated PMPs were kept for protein analysis using the BCA protein assay [20].

PSA capture antibody 20–80 µL (46–184 µg) was added to the remaining goat anti-mouse conjugated PMPs and the solution was then incubated at room temperature overnight with continuous mixing. The PMPs were then washed ×2 in phosphate buffered saline (PBS), pH 7.2 and resuspended in 0.5 mL of storage buffer, 0.1 M PBS/0.1% glycine/0.1% sodium azide. The supernatant, washes and 50 µL of the goat anti-mouse/monoclonal antibody conjugated PMPs were kept for analysis in the BCA protein assay (Pierce Chemical Company, Rockford, IL, USA). Fifty microlitres of 10% sterile BSA was then added to the conjugate to produce a final concentration of 1% BSA for storage purposes.

#### 2.4.2. Preparation of Nitrocellulose Membranes

Test and control lines were prepared using nitrocellulose membrane HF07504 (Millipore Corporation, Billerica, MA, USA) which has a nominal pore size of 13–17 μm. Two antibody lines were deposited onto each membrane. The deposition of antibody lines was undertaken using mechanical deposition using the BioDot AD 3200. Antibody dilutions were prepared in PBS containing 1% BSA.

After antibody deposition, membranes were blocked in PBS/1% BSA for 20 min, washed three times in PBS and soaked in PBS for at least 30 min. The glass fiber conjugate pad (Millipore Corporation, Billerica, MA, USA) was also blocked in PBS/1% BSA for 20 min prior to use.

Once blocked in PBS/1% BSA, the lateral flow assay was constructed by hand as follows, membranes were placed onto backing card (Schleicher & Schuell, Dassel, Germany) containing a layer of adhesive which secured the prepared nitrocellulose membrane in position. The adhesive layer was protected by a layer of waxed paper which could be removed in sections to enable accurate positioning of the lateral flow assay components. The blocked conjugate pad and the absorbent pad (Millipore Corporation, Billerica, MA, USA) were placed on the backing card after removal of additional sections of the waxed paper so that they overlapped the nitrocellulose membrane by 1–2 mm. The prepared lateral flow material was marked into 0.9 cm widths and cut into strips using a sharp pair of scissors.

#### 2.4.3. Lateral Flow Assay

A range of total PSA96/670 (PSA^T^) concentrations 300 to 0.01 ng/mL were prepared by dilution in PBS/1% BSA/0.05% Tween 20, a different dilution was applied to each lateral flow assay strip. In each assay, 5 μL of PMP conjugate containing approximately 5 × 10^6^ PMPs was added to the conjugate pad followed immediately with 60 µL of PSA^T^ standard. The assay was stopped after 5 min once all visible conjugate had flowed through the membrane past the test and control lines by removal of the wicking pad.

Multiple lateral flow assays for total PSA were performed (n = 3) and measurements at the control and test capture lines were made using the RCM and scanning densitometer (Shimadzu Dual Wavelength Flying Spot Scanning Densitometer, Manchester, UK) in comparison to control membrane without PMPs.

#### 2.4.4. Scanning Densitometer

An initial wavelength scan performed with PMPs identified the optimum absorbance for PMPs to be 380 nm, therefore all lateral flow strips were scanned at this wavelength. The peak height values at test and control lines were measured for each LF assay strip.

#### 2.4.5. RCM

The voltage response was logged and the change in voltage was determined by Equation (2). As described in [14].

Change in voltage (mV) = V_1_ − V_2_ (2)

where V1 is the average voltage of reading taken every ten seconds for 100 s for each membrane without PMPs and V2 is the average voltage reading taken every ten seconds for 100 s with PMPs at the test line or control line.

#### 2.4.6. Data Manipulation

The peak height values measured by the scanning densitometer and the change in voltage (mV) recorded by the RCM for the test and control lines was then expressed as a ratio for each LF assay strip and was plotted against PSA concentration (ng/mL). The use of test to control line ratios has advantages for use in quantitative lateral flow assays in that the influence of any factor which affects the PMP flow rate on any individual assay strip is minimized [21]. This process also allowed comparison of data obtained with the same LF assay strips measured using the scanning densitometer.

#### 2.4.7. Statistics

All dose response plots were analyzed by regression analysis and the coefficient of determination, R^2^ was also obtained [22]. The coefficient of determination was calculated using data from replicate assays and assay sensitivity was determined from the slope of the dose response plots. The data obtained from the lateral flow assay measured using the RCM and the FastPack assay were compared with the data obtained from the Immulite assay (DPC, West Sacramento, CA, USA) to determine agreement (Kappa coefficient), diagnostic accuracy and associated sensitivity and specificity [23,24,25].

## 3. Results and Discussion

**Figure 3:**
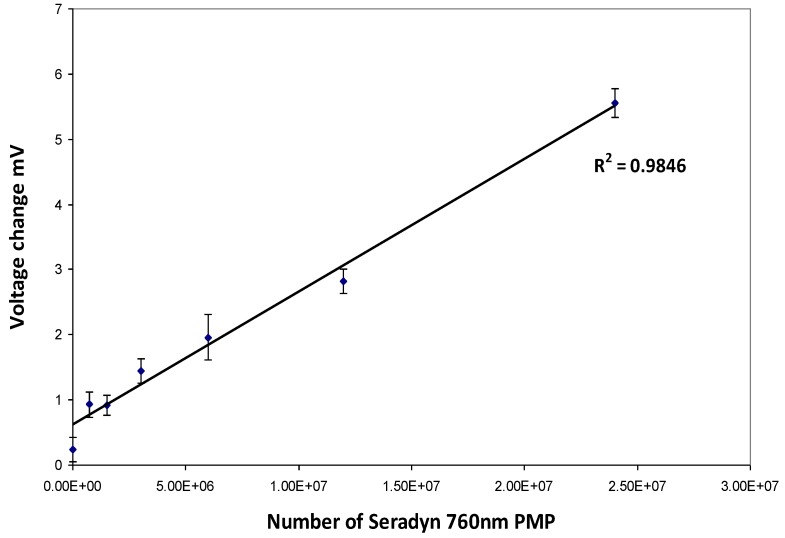
Relationship of number of Seradyn 760 nm paramagnetic particles (PMPs) and response in the RCM Slope = 2.044 × 10^−7^ mV/PMP.

A linear relationship (R^2^ = 0.984) between number of Seradyn 760 nm PMP and change in voltage (mV) was observed using the RCM see Figure 3, with a sensitivity of 0.024 mV/10^5^ PMP (slope = 2.04 × 10^−7^ mV/PMP).

Using the definition provided by the International Union for Pure and Applied Chemistry (IUPAC) the limit of the SM RCM ×2 the standard deviation of the negative control was 1.5 × 10^6^ PMPs.

### Lateral Flow Assays for Total PSA

**Figure 4:**
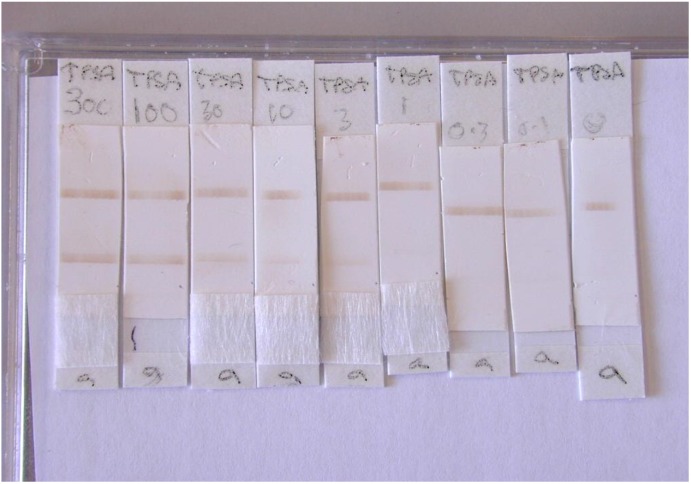
Photograph of the total PSA lateral flow assay.

**Figure 5:**
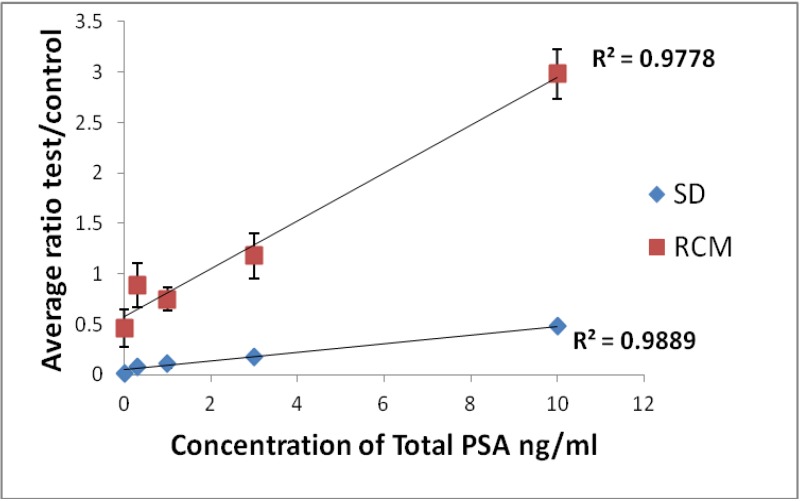
Dose response for total PSA expressed as the average ratio of test to control line by scanning densitometer (SD) (peak height measurements at 380 nm) and by RCM (change in voltage mV) n =3.

A photograph showing the brown test and control lines produced by the captured PMPs in the lateral flow assay for at a range of concentrations of PSA^T^ is shown in Figure 4. The voltage change at the test and control line for each concentration of total PSA was determined. Results were expressed as the mean ratio of the test/control line and were plotted against PSA concentration in ng/mL, see Figure 5. A linear response was observed for total PSA assays over the clinically relevant range 0.8–10 ng/mL with a limit of detection based on the IUAPC definition ×2 standard deviations of the negative control of 0.8 ng/mL. This is equivalent to previous data obtained for the quantification of PSA in solution using the RCM [26]. The graph shows a comparison of measurements made by optical means and using the RCM. The variability in individual data values (see standard deviation error bars) was much greater in the RCM measurements than in the scanning densitometer measurements. This probably relates to the positioning of the test and control lines in relation to the sensor coils. The sensitivity to changes in concentration of PSA^T^ was greater using the RCM, this may be because this device quantifies all PMPs present within the entire test or control lines in contrast to the scanning densitometer which detects only those PMPs present at the surface of the strip. For PSA^T^ quantification the sensitivity of the assay is very important, as even a modest increase in PSA^T^ concentration over time can be indicative of disease progression [27].

The linear range observed was narrower than in a number of commercially available immunoassays, as was the limit of detection [28]. These assays are performed in solution, are commercially produced and run generally on large hospital analyzers, in comparison to the handmade single lateral flow assays described in this study. Further investigation of different particle and membrane combinations, covalent conjugation of antibody, concentration of antibody [26] and optimization of the magneto-immunoassay method would extend both of these assay characteristics. A narrow dynamic range has been observed in many other lateral flow assays; however this has been successfully extended by using alternative labels [29].

- Reproducibility

The results obtained are shown in Table 1. The coefficient of variation (CV) ranged from 39.7 for the control to 8.3 for 10 ng/mL PSA^T^. High variation in the CV of the control without PSA^T^ relates in part to the low signal in the absence of PMPs and the noise of the RCM at these voltages.

**Table 1:** Table showing the results obtained in the lateral flow assay measured by RCM expressed as the mean ratio of the test and control line measurements.

PSA^T^ (ng/mL)	Mean ratio Test/Control	Standard deviation	Coefficient of variation
**30**	3.18	0.321	10.1
**10**	2.98	0.247	8.3
**3**	1.18	0.222	18.8
**1**	0.75	0.113	15.0
**0.3**	0.89	0.216	24.3
**0**	0.46	0.183	39.7

Use of alternative buffers, alternative blocking agents and stabilizers may enable the PMPs to flow through the membrane more reproducibly [30]. The wicking pad influences the speed of flow and therefore the amount of time the conjugate is in close proximity with the capture antibody. Many different wicking pads are available at different thickness and this may make results more reproducible. The limit of detection may also be altered by these modifications; wicking pads that act more efficiently would effectively increase the capillary flow rate of the PMP in the membrane and therefore alter the time that the PMP conjugate and capture antibody are in close proximity. The conjugate and absorbent pads were used at fixed dimensions, however they were cut manually using scissors and some variability may have been introduced as a result. In addition, the degree of overlap between the various components of the lateral flow assay (normally 1–2 mm) can have a significant effect on the flow rate [31]. This issue can be overcome by precise mechanical construction of the lateral flow assay strips.

The impact of factors that influence the flow rate of PMPs in individual assay strips were minimized by use of the ratio of the test to the control line as described earlier. Other possible influences are factors which relate to the conjugate. Inconsistencies in passive conjugation or deterioration in the conjugate over time require investigation as to their effect on assay variability. Further investigation of both passive and covalent conjugation with PMP of a range of sizes may improve reproducibility. The conjugate containing glycine and BSA is kept in storage buffer at 4 °C, the rate and efficiency of flow of the conjugate may vary with age of the PMP conjugate. The conjugate was sonicated and efficiently resuspended before use in each experiment, but as PMPs tend to aggregate over time, a more effective storage buffer may reduce aggregation and potential effects on flow rate.

Commercially available lateral flow assays are produced where the humidity and temperature are controlled at each step of the manufacturing process. The preparation of the membranes and lateral flow assay strips at room temperature and at sub-optimal humidity may result in an uneven flow of liquid in lateral flow assays which will result in some variability in results [32].

Variability in the RCM data may relate to the manual positioning of the capture lines in relation to the inductive sensor of the RCM. Previous studies have shown that this is critical to obtain reproducible data [33] and this may be compounded in the present RCM design as the magnetic field from the surface mounted coils does not extend more than a few millimeters. Accurate positioning of the capture lines using a mechanical locator will therefore be incorporated in future studies.

- Diagnostic Accuracy of Immuoassays in Comparison to the Immulite Assay

The standard curve for PSA^T^ (96/670) used to determine the concentration of PSA^T^ in twenty five serum samples is shown in Figure 5. The results obtained in the lateral flow assay measured using the RCM were compared to those obtained in the FastPack assay in comparison to the Immulite assay, the results are presented in Table 2. These results were determined using positive and negative predictive values based on a cut-off value of 4 ng/mL.

**Table 2:** Diagnostic accuracy of FastPack and magneto-immunoassay (RCM) in comparison with the Immulite assay analysed by methods described by Cohen [23] and Altman and Bland [24,25].

Immunoassay	Positive test results			Negative test results			Sensitivity	Specificity	κ
	True positive	False positive	PPV	True negative	False negative	NPV			
FastPack	8	1	88%	15	0	100%	100%	93.8%	0.91
RCM	9	4	69%	15	0	100%	100%	79%	0.71

**Key:** TP = True positive, FP = False positive, TN = True negative, FP = False positive; PPV = Positive predictive value is the probability that a test positive is a true positive (PPV = TP/(TP + FP)); NPV = Negative predictive value is the probability that a test negative is a true negative (NPV = TN/(FN + TN)); Sensitivity = TP/(TP+FN) Sensitivity is the proportion of true positives that are correctly identified by the test; Specificity = TN/(FP+TN) Specificity is the proportion of true negatives that are correctly identified by the test. κ = Ƙappa coefficient, indicator of agreement = PQ + P*'*Q*'*/S. P = TP + FN, P*'* = FP + TN, Q = TP + FP, Q*'* = FN + TN, S = P + P*'*.

The results show that the two commercial assays are in generally very good agreement, the Fastpack PSA assay gave a Kappa coefficient of 0.91, however some differences were observed. The magneto-immunoassay demonstrated good agreement with a kappa coefficient of 0.71 and excellent sensitivity of one hundred percent; however the specificity was 85.7% as four false positives (*i.e*., >4 ng/mL) were detected. All false positives were positive for PSA^T^ in the two commercial assays, but the concentrations of PSA^T^ detected in these immunoassays were below 4 ng/mL. The magneto-immunoassay has therefore overestimated the concentration of PSA^T^ in comparison to the commercial assays. Differences in the measurements obtained for the NIBSC 97/680 preparation of PSA^T^ between immunoassays are consistent with other studies [34] and may relate in part to the specificity and affinity of the antibodies used. It is possible that heterophilic antibodies present in the serum may also have elevated the positive values in some samples [35] and evaluation of reagents to minimize these effects is essential. The results may also have been influenced by the coefficient of variation observed in the magneto-immunoassay as described earlier and may be improved by the mechanical preparation and positioning of the lateral flow assay strips. Assay variability in experimental quantitative lateral flow assays has been observed by others and indicates the challenge of using immunochromatography to provide quantitative data under research conditions [36].

## 4. Conclusions

In this investigation the aim of the study was to confirm that PMPs can be conjugated, used in lateral flow and that PMPs collected at capture lines can be quantified using the RCM.

Using a standard PSA preparation, a magneto-immunoassay was developed to clinically relevant limits of detection (0.5–10 ng/mL) with numbers of PMPs bound at test and control lines quantifiable using the RCM by virtue of the magnetic properties of the PMPs.

The assays developed were optimized to confirm detection of PMPs captured at test and control lines in lateral flow assays by the RCM *i.e*., with the greatest number of PMP bound at test and control lines. Improvements in assay limit of detection, linear range, sensitivity and reproducibility will be achieved through further investigation of levels of antibody coating of PMPs, methods of PMP conjugation and mechanical construction of lateral flow assay strips. Further development of the RCM to detect lower numbers of particles and mechanical positioning of the test and control lines within the RCM will also assist in the improvement in assay sensitivity and characteristics.

New developments in lateral flow assay materials [3] may also provide greater reproducibility in results which have so far limited the use of this technology for quantitative assessments.

Multi-analyte lateral flow assays using latex or gold are already used and this could therefore be applied to magneto-immunoassays. The measurement of free and total PSA as a ratio has been reported to be more predictive of prostate cancer [37]. In addition, several other analytes have been proposed as potential markers to aid in the diagnosis and prognosis of prostate cancer in particular other members of the Kallikrein family of proteins [38]. The inclusion of new biomarkers [39] in lateral flow in series or in parallel that correlate with the presence of prostate cancer to produce a profile, may lead to improvements in the diagnosis and prognosis of prostate cancer. The RCM design described could be modified to allow for colocation of duplicate FLL circuits which would enable multi-analyte quantitation.

This investigation shows promise for use of the RCM as an affordable device to quantify multiple analytes in lateral flow, at the point-of-care in a clinic or doctor’s surgery.
